# Circulating serum fibroblast growth factor 21 as risk and prognostic biomarker of retinal artery occlusion

**DOI:** 10.1038/s41598-024-62588-w

**Published:** 2024-05-24

**Authors:** Ruobing Shi, Hang Liu, Kaichao Xia, Ying Li, Ting chen, Xuejie Li, Anhuai Yang, Xuan Xiao

**Affiliations:** 1 Department of Ophthalmology, Renmin Hospital of Wuhan University, No. 238 Jiefang Road, Wuhan, Hubei Province, 430060 China; 2 Department of Clinical Laboratory, Institute of Translational Medicine, Renmin Hospital of Wuhan University, Wuhan, China

**Keywords:** Fibroblast growth factor 21, Retinal artery occlusion, Visual acuity improvement, Ischemic stroke, Stroke, Biomarkers, Risk factors

## Abstract

To evaluate the predictive and prognostic value of fibroblast growth factor 21 (FGF21) levels in retinal artery occlusion (RAO) patients. In this case–control study, serum FGF21 levels were detected by using the ELISA method. Multivariable logistic regression analyses were performed to evaluate the significance of FGF21 in assessing the risk of developing RAO and its impact on vision and concurrent ischemic stroke. Compared with control group, serum FGF21 levels were significantly higher (median [IQR] = 230.90[167.40,332.20] pg/ml) in RAO patients. Multivariate logistic regression analysis showed that elevated serum FGF21 levels were associated with a higher risk of RAO occurrence (*P* = 0.025, OR [95%CI] = 9.672 [2.573, 36.359]) after adjustment for multiple confounding factors. Higher serum FGF21 levels were negatively associated with visual acuity improvement (*P* = 0.029, OR [95%CI] = 0.466[0.235, 0.925]) and positively correlated with concurrent ischemic stroke (*P* = 0.04, OR [95% CI] = 1.944[1.029, 3.672]) in RAO patients. Elevated serum FGF21 levels could promote the development of RAO and indicate worse visual prognosis and increase the risk of concurrent ischemic stroke, which might help clinicians early diagnose and treat RAO patients.

## Introduction

Retinal artery occlusion (RAO) is an ophthalmic emergency characterized by instant and severe visual impairment^1^. According to a consensus statement published by The American Heart Association, RAO is defined as a variant of acute ischemic stroke^2^. Though the estimated incidence of acute CRAO is 2–3 per 100,000^3^, the majority of patients experience a visual prognosis below functional visual acuity and are at an elevated risk for stroke^3,4^, posing a significant threat to aged health. Currently, the diagnosis of RAO primarily relies on clinical manifestations of monocular vision loss and typical funduscopic findings of retinal edema and cherry red spot^3^, without additional existing specific tools for early prediction and diagnosis. Thus, earlier detection of RAO patients becomes crucial. Several approaches have been developed to treat RAO, including tPA administration, anterior chamber paracentesis and carbogen therapy^2^. However, seldom of them have exhibited excellent efficacy based on current clinical evidence.

The development of RAO has been closely associated with a high prevalence of cardiovascular risk factors and metabolic disease^2^. Our previous research has demonstrated that diabetes increases the risk of cardiovascular and cerebrovascular events in patients with RAO^5^. Besides, diabetes could induce retinopathy through vascular injury, remodeling triggered by hyperglycemia, and direct impact on retinal neural and glial tissue^6,7^. The latest research indicates that fibroblast growth factor 21 (FGF21) has important roles in regulating energy balance and glucose and lipid homeostasis^8^. It has been identified as a potential biomarker in multiple metabolic disorders such as type 2 diabetes and cardiovascular disease^9,10^, and it can also improve neurological outcomes in ischemic stroke mice and anti-atherogenic effects in vitro^11,12^. Furthermore, FGF21 analogs are emerging therapeutic targets for type 2 diabetes and diabetic retinopathy^13,14^. These available evidences suggest that the intricate mechanisms of FGF21 may play an important role in the pathogenesis and prognosis of RAO.

Previous studies mainly focused on the effects of FGF21 and cerebral stroke, however, the relationship between FGF21 and ocular stroke—RAO has not been fully studied. Thus, this study aimed to investigate the alterations of FGF21 levels among RAO patients and explore the prospective association between FGF21 levels and both the occurrence and prognosis of RAO. Our findings may lay the foundation of FGF21 for the early diagnosis and treatment and prognosis assessment of RAO.

## Materials and methods

### Patient selection

RAO patients were admitted to Renmin Hospital of Wuhan University from January 2020 to November 2023. This research was conducted as a retrospective comparative case series, following the guidelines of the Declaration of Helsinki, and received approval from the institutional review board of the Renmin Hospital of Wuhan University (Wuhan, China, WDRY2022-K278). Since this study is retrospective and does not involve patient privacy information, the Ethics Committee of Renmin Hospital of Wuhan University waived the requirement of patients' informed consent.

**Figure 1:**
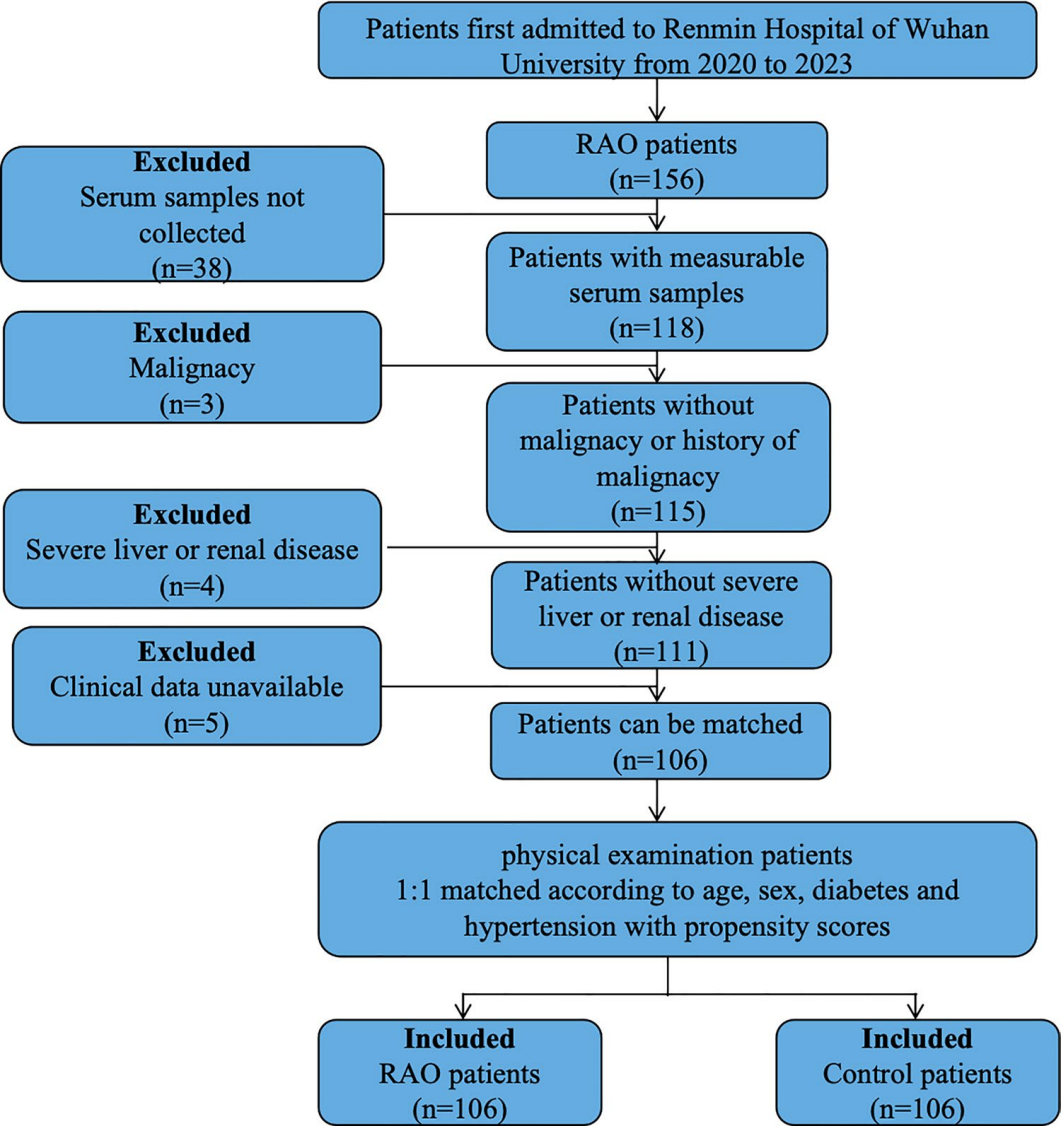
Flowchart of Inclusion and Exclusion criteria of participants.

The inclusion and exclusion criteria flowchart of control participants and RAO patients was drawn in Fig. 1. The diagnosis of RAO was based on clinical manifestation and funduscopic findings in accordance with the guidelines (Supplementary Figs. 1 and 2)^3,15^. A total of 106 consecutive patients with a first diagnosis of spontaneous non-arteritic RAO were included. To rule out confounding factors such as gender, age, diabetes and hypertension, the control group consisted of randomly selected patients, which were matched with the study group based on the above factors at a ratio of 1:1 using propensity score matching (PSM). The baseline demographics were obtained from patient medical records. Hypertension patients were defined as individuals who had a record of hypertension history, using antihypertensive medications within the past 2 weeks, or with systolic blood pressure (SBP) ≥ 140 mmHg or diastolic blood pressure (DBP) ≥ 90mmHg^16^. Patients who had a record of diabetes history, taking hypoglycemic drugs or whose measured blood glucose exceeded the guidelines were considered as diabetes patients^17^.

### FGF21 measurement

Blood samples were obtained from participants after a 12-h period of fasting using the standard technique for venipuncture. FGF21 levels were measured in serum samples using a sandwich enzyme linked immunosorbent assay kits (R&D systems, Minneapolis, USA, DY2539) with streptavidin-HRP, hydrogen peroxide and tetramethylbenzidine included in the color development system. Intra-assay and inter-assay coefficients of variation were 2.9–3.9% and 5.2–10.9% respectively. We meticulously constructed a standard curve for each experiment according to the manufacturer's instructions, ensuring that only when the R-squared value exceeded 0.999 could it fulfill our subsequent analysis requirements (Supplementary Fig. 3).

### Laboratory measurement

The blood counts and biochemical indexes were measured by venous blood samples. Using automatic blood cell analyzer XN-9000 (Sysmex Corporation, Kobe, Japan) to measure the counts of white blood cells (WBC), neutrophils (Neu), lymphocytes (Lym) and monocyte (Mono). Using automatic biochemical analyzer Advia 2400 (Siemens Medical Solutions, Erlangen, Germany) to measure the levels of triglycerides (TG), total cholesterol (TCh), low-density lipoprotein cholesterol (LDL-Ch), high-density lipoprotein cholesterol (HDL-Ch), glutamic pyruvic transaminase (ALT), glutamic oxaloacetic transaminase (AST), glucose (Glu) and urea (Urea). The estimated glomerular filtration rate (eGFR) was calculated using the CKD-EPI Creatinine formula^18^.

### Visual outcome measurement

All patients were evaluated by an experienced ophthalmologist at admission and before discharge. The assessment of visual acuity (VA) was performed using a standard logarithmic visual acuity chart, and the results were converted to logarithm of the minimum angle of resolution (logMAR) based on the formula logMAR = lg(1/decimal VA) ^19^. Additionally, for low vision categories such as "counting fingers", "hand motion", "light perception" and "no light perception," corresponding logMAR values were determined as 2.0, 2.3, 2.6, and 2.9 respectively^20^. A favorable visual outcome was defined as a significant improvement in visual acuity, characterized by a reduction of 0.3 or more in logMAR^21^.

### Concurrent ischemic stroke measurement

The primary outcome was occurrence of a concurrent ischemic stroke. All patients underwent cerebral magnetic resonance imaging (MRI) within 7 days of RAO onset and all images were evaluated by a certified neurologist for the presence of acute ischemic lesions. MRI was performed on 1.5 T scanners (GE Signa; GE Medical Systems, Milwaukee, Wisconsin). High signal intensity on diffusion-weighted imaging (DWI) and low signal intensity on the apparent diffusion coefficient (ADC) maps, or low signal intensity on T1-weighted after 16 h of initial stroke and high signal intensity on T2-weighted after 8 h of initial stroke were considered acute ischemic lesions^22^.

### Statistical analysis

Continuous variables were described as median and interquartile range. The group comparisons of continuous data were conducted by the Mann–Whitney test. Categorical variables were presented as numbers and percentages. Chi-square tests were conducted for comparison of categorical data. The association between ln-transformed serum FGF21 levels and the occurrence of RAO and its visual and concurrent ischemic stroke outcomes was assessed by logistic regression analysis. The correlates of serum FGF21 levels were assessed by multivariable linear regression analysis. The potential nonlinearity relationship between FGF21 levels and the occurrence of RAO was assessed using restricted cubic regression splines in R software.

Statistical analysis was conducted by SPSS 26 (IBM, Armonk, NY) and R 4.2.3 (The R Core Team, Vienna, Austria). A two-sided *p* value < 0.05 was considered statistically significant.

## Results

### Baseline characteristics of RAO patients

The characteristics of control group and RAO group were summarized in Table 1. In terms of clinical data, there were no statistically significant differences in gender, age, proportion of diabetes patients and hypertension patients between the two groups after propensity score matching. The prevalence of carotid artery stenosis or plaque in RAO patients was significantly higher compared to the control group. In terms of laboratory indicators, FGF21 levels in the RAO group were significantly higher than in the control group (230.90 [167.40, 332.20] pg/ml versus 153.60 [77.35, 244.40] pg/ml, *p* < 0.001). Additionally, RAO patients presented higher levels of neutrophil, monocyte, NLR, TG and Glu (all *p* < 0.01), while lymphocyte, HDL-Ch and eGFR levels were lower (all *p* < 0.05). There were no differences in WBC, ALT, ALT/AST, Tch, LDL-Ch and Urea between the two groups.

**Table 1:** Baseline characteristics of control group and RAO group.

Characteristics	Control group n = 106	RAO group n = 106	*P* value
Clinical variables			
Male sex (%)	73.58	72.64	0.877
Age (years)	58(55,63)	59(52,68)	0.302
Hypertension (%)	48.11	50.94	0.680
Diabetes (%)	6.60	7.55	0.789
Carotid artery stenosis (%) (or plaque)	6.60	50.94	< 0.001
Heart disease (%) (atrial fibrillation, valvular disease, or coronary heart disease)	3.77	5.66	0.748
Laboratory variables			
WBC (× 10^9^/L)	5.79(4.86,6.89)	6.24(5.08,7.39)	0.092
Neu (× 10^9^/L)	3.10(2.57,3.68)	3.53(2.81,4.56)	0.001
Lym (× 10^9^/L)	2.01(1.66,2.48)	1.77(1.45,2.29)	0.015
NLR	1.44(1.18,1.98)	1.99(1.45,2.52)	< 0.001
Mono (× 10^9^/L)	0.42(0.33,0.52)	0.49(0.38,0.59)	0.003
ALT (U/L)	18.00(15.00,25.00)	17.00(13.00,24.50)	0.346
AST (U/L)	22.00 (18.00,25.00)	19.00 (16.00,24.00)	0.003
ALT/AST	0.88 (0.71,1.08)	0.95(0.68,1.20)	0.164
TCh (mmol/L)	4.54(4.12,4.86)	4.51(3.84,5.19)	0.584
TG (mmol/L)	1.11(0.95,1.34)	1.51(1.00,1.90)	< 0.001
HDL-Ch (mmol/L)	1.23(1.12,1.44)	0.98(0.85,1.18)	< 0.001
LDL-Ch (mmol/L)	2.62(2.29,2.97)	2.80(2.23,3.33)	0.082
Urea (mmol/L)	5.31(4.63,6.02)	5.44(4.64,6.51)	0.200
eGFR (ml/min/1.73m^2^)	97.93(93.79,101.80)	93.70(84.18,104.50)	0.026
Glu (mmol/L)	4.82(4.47,5.16)	5.25(4.87,5.89)	< 0.001
FGF21 (pg/ml)	153.60(77.35,244.40)	230.90(167.40,332.20)	< 0.001

Data are expressed as percent (n) or median (interquartile range).

*WBC*: White blood cell; *Neu*: Neutrophil; *Lym*: Lymphocyte; *NLR*: Neutrophil-to-lymphocyte ratio; *Mono*: Monocyte; *ALT*: Glutamic pyruvic transaminase; *AST*: Glutamic oxaloacetic transaminase; *ALT*/*AST*: Glutamic pyruvic transaminase-to -glutamic oxaloacetic transaminase ratio; *TCh*: total Cholesterol; *TG*: Triglycerides; *HDL*-*Ch*: High-density lipoprotein cholesterol; *LDL*-*Ch*: Low-density lipoprotein cholesterol; *eGFR*: Estimated glomerular filtration rate; *Glu*: Glucose.

**Figure 2:**
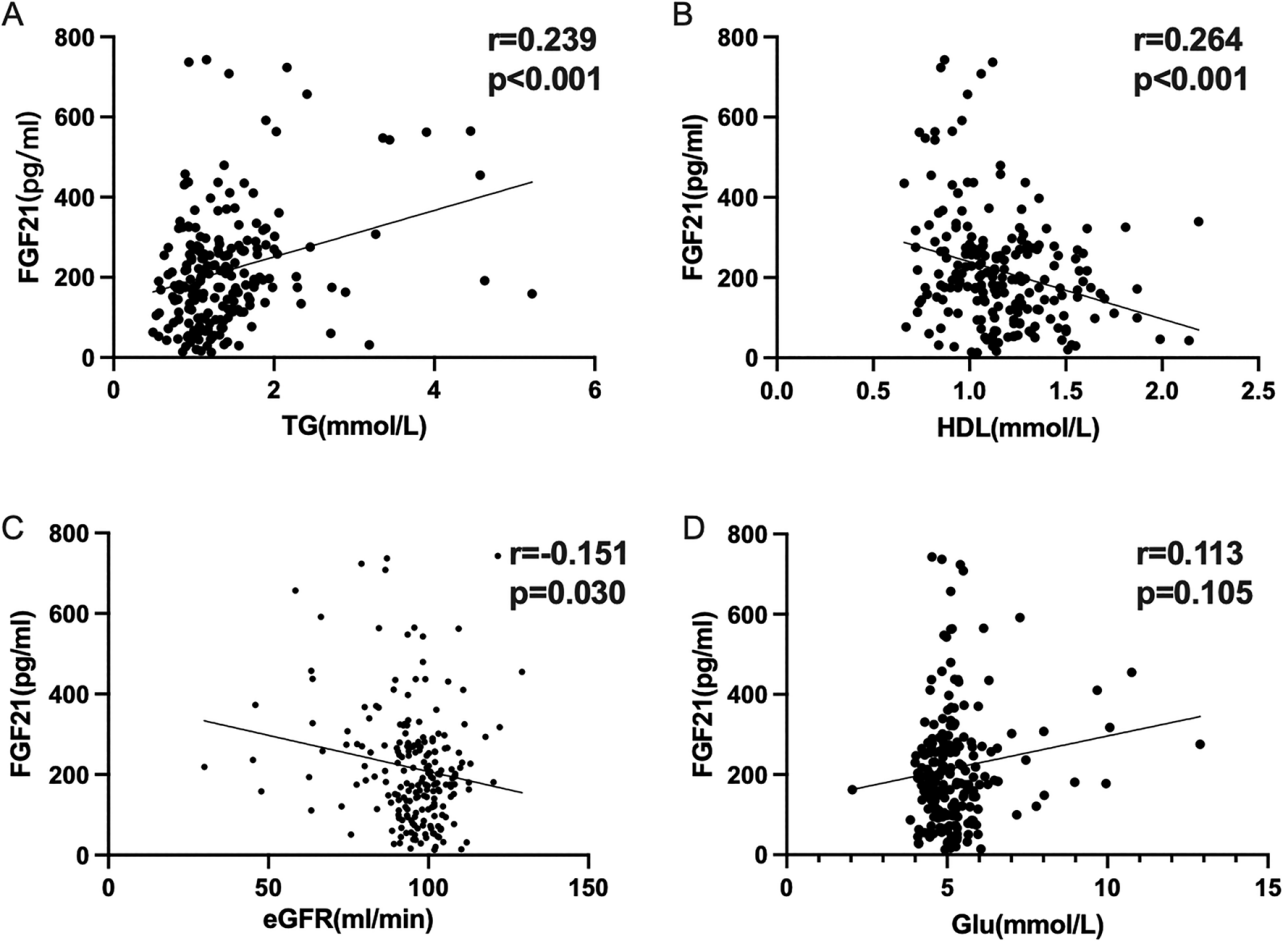
Correlates of FGF21 levels in multivariable linear regression analysis. (**A**) Correlate of FGF21 with TG; (**B**) Correlate of FGF21 with HDL; (**C**) Correlate of FGF21 with eGFR; (**D**) Correlate of FGF21 with Glu. *TG*: triglycerides; *HDL-Ch*: high-density lipoprotein cholesterol; *eGFR*: estimated glomerular filtration rate; Glu: glucose.

The association between each covariate and FGF21 levels was illustrated in Supplementary Table 1. In the multivariable model, FGF21 levels were positively linked with TG, and were negatively linked with HDL and eGFR, which were consistent with the results of Spearman's correlation analysis in Fig. 2. In addition, there was no significant correlation between FGF21 levels and Glu levels in either multivariate linear regression analysis or Spearman's correlation analysis.

### Association between the prevalence of RAO and FGF21

To further analyze the correlation between FGF21 and risk of RAO, the FGF21 levels were divided into quartiles, taking the first quartile as the reference to evaluate the odds ratio for RAO (Table 2). FGF21 presented a positive association with the prevalence of RAO in the crude model and mode l (adjusted for clinical data such as sex, age, diabetic and hypertension). After additional adjustment for Neu, Lym, NLR, Mono, AST, TG, HDL, eGFR and Glu in model 2, this correlation remained statistically significant. In the complete adjustment model 2, the OR (95%CI) was 9.672 (2.573, 36.359) for quartile 4 of FGF21 levels (the highest) versus quartile 1 (the lowest). Similarly, after adjusted confounding factors including Neu, Lym, NLR, Mono, AST, TG, HDL, eGFR and Glu, the association of FGF21 with RAO development is still significant (Supplementary Table 2).

**Table 2:** Logistic regression analysis of ln-transformed serum FGF21 level for RAO.

FGF21 quartile	N	FGF21 (pg/ml)	OR(95%CI)		
			Crude	Model 1	Model 2
Quartile1	53	≤ 119.561	Reference	Reference	Reference
Quartile2	53	119.561–192.666	4.128(1.782, 9.567)	2.990 (0.909, 9.840)	3.947(1.171, 13.307)
Quartile3	53	192.666–273.765	2.828(1.22, 6.553)	2.759(0.896, 8.496)	3.112(0.952, 10.165)
Quartile4	53	≥ 273.765	11.674(4.7, 28.994)	11.244 (3.168, 39.911)	9.672(2.573, 36.359)
β			2.457	2.420	2.269
SE			0.464	0.646	0.676
P for trend			< 0.001	< 0.001	0.001

Crude: No adjustment.

Model 1: Adjusted for sex, age, hypertension and diabetes.

Model 2: Adjusted for the same variables as Model 1 as well as Neu, Lym, NLR, Mono, AST, TG, HDL, eGFR and Glu.

**Figure 3:**
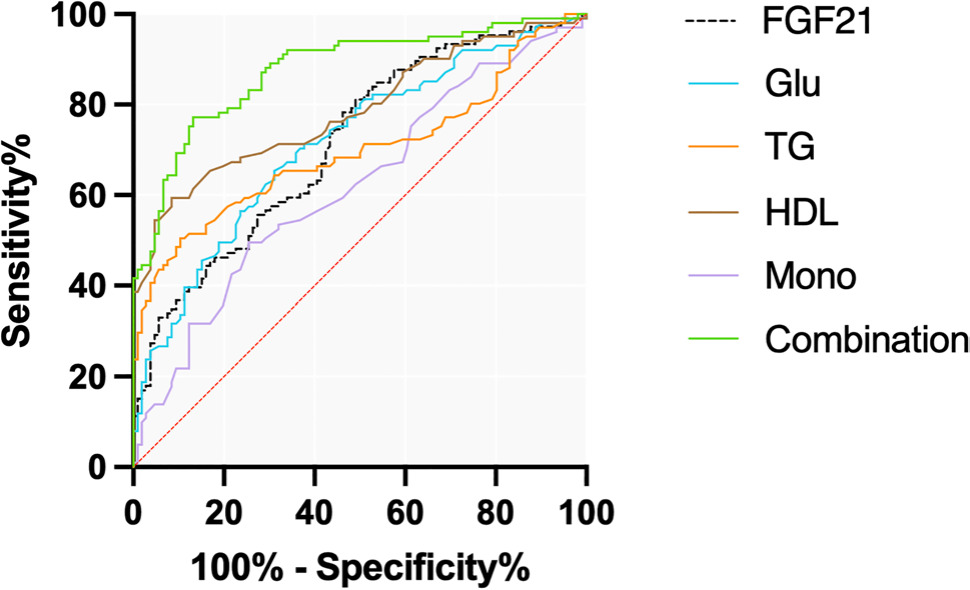
ROC curve analysis of FGF21 combined with laboratory indexes in the diagnosis of RAO. *Glu*: Glucose; *TG*: Triglycerides; *HDL*: High-density lipoprotein cholesterol; *Mono*: Monocyte; *Combination*: FGF21 + glucose + triglycerides + high-density lipoprotein cholesterol + monocyte.

In addition, restricted cubic spline analysis of complete adjusted data also indicated a positive correlation between the prevalence of RAO and serum FGF21. As shown in Fig. 3, the curve illustrated a threshold value at approximately 5.26 in ln-transformed FGF21 levels (equivalent to an FGF21 level of 192.67 pg/mL) for predicting RAO risk, beyond which the odds ratio increases.

### ROC analysis of predictive value of FGF21

**Figure 4:**
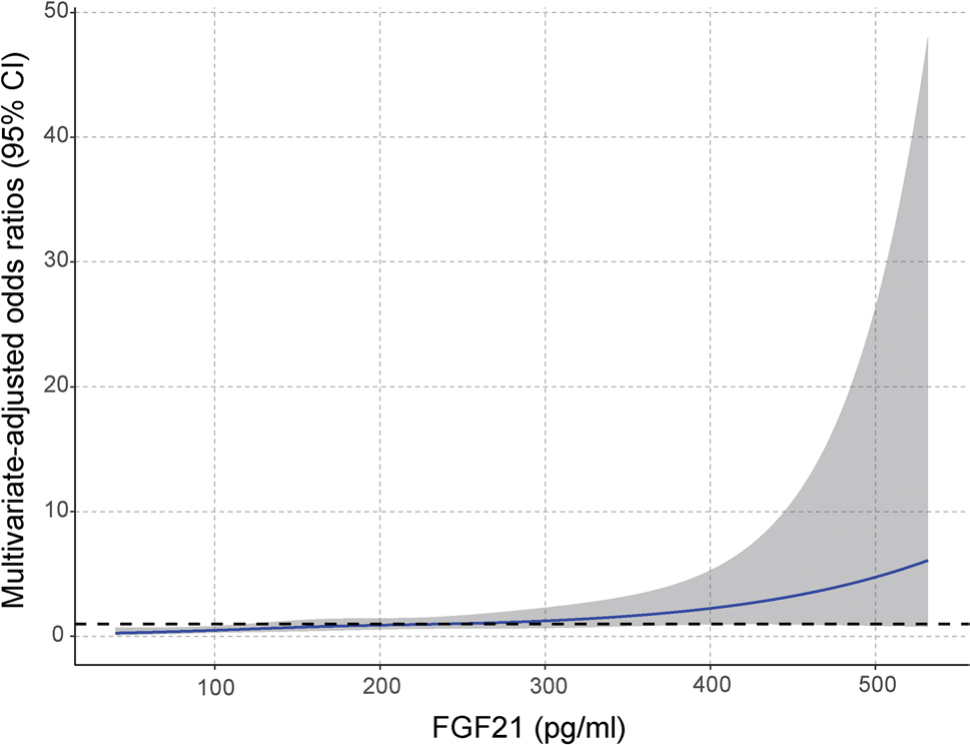
Restricted cubic spline model of the odds ratios of RAO with serum FGF21 levels.

To evaluate the value of FGF21 in predicting the occurrence of RAO, we performed ROC analysis and compared with other laboratory indicators. As shown in Table 3 and Fig. 4, the area under the curve (AUC) of FGF21 was 0.715 ([95% CI] = [ 0.647, 0.783]), and the sensitivity was 81.13%, which was the highest among all indicators analyzed above, and the specificity was 50.09%, the Youden index was 0.3207. There indicated that FGF21 may possess a degree of accuracy in forecasting probability of RAO occurrence.

**Table 3:** ROC curve analysis of FGF21 combined with laboratory indexes in the diagnosis of RAO.

Variables	AUC	Sensitivity%	Specificity%	95%CI	Youden index	Cut-off
FGF21	0.715	81.13	50.09	0.647, 0.783	0.3207	156.70 pg/ml
Glu	0.716	65.35	68.87	0.647, 0.786	0.3422	5.08 mmol/L
TG	0.699	50.50	89.62	0.625, 0.773	0.4012	1.50 mmol/L
HDL	0.787	59.41	91.51	0.725, 0.850	0.5092	1.03 mmol/L
Mono	0.621	49.50	74.53	0.545, 0.697	0.2403	0.50 × 10^9^/L
Combination	0.877	77.23	86.79	0.829, 0.925	0.6402	—

*Glu*: Glucose; *TG*: Triglycerides; *HDL*: High-density lipoprotein cholesterol; *Mono*: Monocyte; Combination: FGF21 + glucose + triglycerides + high-density lipoprotein cholesterol + monocyte.

### Association between FGF21 and visual and concurrent ischemic stroke outcomes in RAO patients

As summarized in Table 4, there was a negative correlation between ln-transformed FGF21 levels and VA improvement (OR [95% CI] = 0.466[0.235,0.925], *P* = 0.029) after adjusted for age, gender, hypertension, diabetes, treatment and initial VA. However, the association lost statistical significance upon stratified analysis based on FGF21 levels. In terms of the percentage of visual acuity (VA) improvement, the trend of decreased percentage with increasing FGF21 levels remained consistent.

**Table 4:** Association of baseline ln-transformed plasma FGF21 levels with ischemic stroke and visual improvement in RAO patients.

Event	No. of events, % (n)	OR (95%CI)	*P*
Ischemic stroke			
ALL	54.72(58)	1.944(1.029,3.672)	0.040
FGF21 < 192.67	46.34(19)	1.107(0.367,3.336)	0.857
FGF21 ≥ 192.67	60.00(39)	6.597(1.323,32.893)	0.021
Visual improvement			
ALL	35.58(37)	0.466(0.235,0.925)	0.029
FGF21 < 192.67	42.50(17)	0.381(0.094,1.552)	0.178
FGF21 ≥ 192.67	31.25(20)	0.289(0.058,1.433)	0.129

The correlation between FGF21 and the incidence of ischemic stroke exhibited a positive association. After adjustment for age, gender, hypertension, diabetes, hyperlipemia, smoking, and alcohol use, the incidence of ischemic stroke was positively correlated with ln-transformed FGF21 levels (OR [95%CI] = 1.944 [1.029,3.672], *P* = 0.04). In stratified analyses according to FGF21 levels, the association remained statistically significant for participants with FGF21 levels ≥ 192.67 pg/mL, but not for those with FGF21 levels < 192.67 pg/mL.

## Discussion

RAO is an ocular stroke that leads to severe, irreversible visual impairment and increased risk of ischemic stroke, currently lacking effective diagnosis and treatment options. In the present study, we found that serum FGF21 levels were elevated in RAO patients. Previous studies have shown that the level of FGF21 was also upregulated in fundus diseases such as diabetic retinopathy and infarct-related conditions like stroke^23,24^. Multivariable logistic regression analysis suggested higher FGF21 levels were associated with RAO and its worse visual and neurological outcomes after adjusting for other confounders. This indicates that FGF21 could be a potential biomarker for the diagnosis and prognosis of RAO.

The levels of FGF21 were found to be elevated in patients with RAO and exhibited significant correlations with TG, HDL, and eGFR. In concordance with previous studies, FGF21 concentrations were positively linked with TG levels, while negatively associated with HDL and eGFR levels^10,25,26^. The prevalence and development of RAO have been reported to be closely linked with cardiovascular risk factors, including hypercholesterolemia^2^. The overexpression of FGF21 could effectively attenuate TG accumulation by inhibiting de novo lipogenesis^27^, and exogenous administration of FGF21 leads to a significant decrease in TG levels and an increase in HDL levels^28^.

Diabetes is also implicated as a predisposing factor for RAO^2^. Previous study found that FGF21 levels positively correlated with Glu in diabetes and impaired glucose tolerance patients^29^, but we did not observe a statistically significant relationship between these two variables in RAO patients. However, we observed significantly higher Glu levels in RAO patients compared to the control group, suggesting a tendency for elevated Glu and an increased likelihood of developing diabetes among RAO patients. This discrepancy may be attributed to variations in glucose tolerance within the study participants. We investigated the association between Glu and FGF21 among individuals, the vast majority of whom had normal glucose tolerance, while the aforementioned study encompassed a broader range of participants including those with impaired glucose tolerance and diabetes. Furthermore, the observation that FGF21 is upregulated in individuals with diabetes is widely acknowledged^9,29,30^. We meticulously balanced the proportion of diabetic patients between the two groups to mitigate the potential confounding effect of diabetes on serum FGF21 levels in RAO patients.

Several studies have consistently reported an elevation of FGF21 in various conditions, including heart disease and metabolic disorders^10,23,29,31,32^. Surprisingly, in animal and in vitro studies, FGF21 confers protection against the above disease conditions. In the heart, FGF21 could reduce cardiac oxidative stress, hypertrophy, and inflammation^33,34^. FGF21 through the mediation of the FGFR1/β-Klotho–PI3K–Akt1–BAD signaling network to reduce cell death, and myocardial infarction thereby ameliorating myocardial function^35^. On the other hand, FGF21 can improve glucose homeostasis in a variety of ways, including stimulates insulin expression and secretion via the PI3K/Akt signaling pathway, protect β cells by activating AMPK-acetyl CoA carboxylase (ACC) and PPARδ/γ signaling pathway, and prevent systemic insulin resistance by increasing adiponectin levels^36^. In fundus disease vivo models, FGF21 prevents pathological neovascularization in the retina and choroid^37^, while also playing a protective role in preserving photoreceptor function in diabetic mice^14^. Moreover, FGF21 administration attenuated the hyperactivity of the intraocular complement system, thereby ameliorating the occurrence and development of age-related macular degeneration ^38^.

In this study, the finding of raised serum FGF21 levels in RAO in humans is paradoxical to preclinical evidence where FGF21 reduced retina hypoxia damage in mice^37,39^. This contradictory phenomenon may be caused by the presence of FGF21 resistance, which was generally considered to result from defects in the downstream signaling pathway of FGF21 receptor complex, leading to high endogenous levels of FGF21^40^. This phenomenon bears resemblance to insulin resistance, as despite the presence of hyperinsulinemia in individuals with type 2 diabetes, insulin therapy continues to exhibit efficacy^41^. Another possible explanation is that FGF21 could potentially be attributed to a compensatory protective response to comorbid metabolic stress which precipitated RAO^2^. The mechanism of FGF21 in RAO closely resembles that observed in the aforementioned diseases, suggesting the potential of FGF21 as a novel predictor and therapeutic target for RAO. Based on the above, we speculate that FGF21 may also play a protective role in RAO. We would further elucidate the molecular function of FGF21 in RAO through additional animal and cell experiments.

RAO is a condition of retinal ischemia secondary to the sequelae of pathological changes of the cardiovascular system, and thus these patients have increased the prevalence and risk of stroke. Acute cerebral infractions were found in 27.0–76.4% of CRAO patients with magnetic resonance imaging with diffusion-weighted imaging^42^, suggesting that these patients warrant urgent stroke assessment. Recent studies showed FGF21 levels are elevated in ischemic stroke and associated with poor prognosis^43,44^. Controversially, FGF21 played a protective role in stroke. In middle cerebral artery occlusion (MCAO) mice model, rhFGF21 through modulating the activation of microglia via inhibiting NF-κB and elevating PPAR-γ, suppresses the inflammatory response^45^. Our study found that high levels of FGF21 were positively correlated with the incidence of ischemic stroke in RAO patients, suggesting that FGF21 levels hold promise as both a predictive marker and a potential therapeutic target for concurrent ischemic stroke management in RAO. Additionally, FGF21 exerts neuroprotective effects by augmenting mitochondrial function through the AMPKα/AKT pathway and attenuating neuroinflammation via inhibition of the NF-κB pathway^46^. The modulation of the PP2A/MAPKs/HIF-1α pathway represents a potential mechanism underlying the beneficial impact of FGF21 on Alzheimer's disease-like pathologies^47^. Our study also discovered that high levels of FGF21 were negatively associated with VA improvement in RAO patients. Since long-acting analogues or agonists of FGF21 hold great promise as potential therapeutic targets for the management of fundus diseases like diabetic retinopathy^14^, as well as neuropathic disease such as Alzheimer’s disease and Parkinson’s disease^46,47^, based on our findings, FGF21 may serve as a viable approach for improving visual outcomes in RAO.

The mechanism underlying the occurrence of retinal artery occlusion (RAO) has not been elucidated. A recent study by Elbeyli, A. et al. suggested that inflammation and oxidative stress may play pivotal roles in the pathogenesis of RAO, with red cell distribution width (RDW) emerging as a superior inflammatory indicator for predicting CRAO^48^. Consistent with this study, our recent study has also identified that increased numbers of neutrophils and T cells could serve as potential inflammation and immune signatures for retinal artery occlusion by using peripheral blood transcriptomic analysis^49^. These studies collectively underscore the multifactorial nature of RAO, implicating both biochemical markers such as FGF21 in this study and inflammatory indices derived from blood count. Integrating these findings into clinical practice could potentially improve risk assessment, early detection, and management strategies for RAO patients.

We thoroughly reviewed the medical records of all participants to confirm cases of RAO and excluded any incorrect or uncertain diagnoses. To maintain homogeneity in terms of ethnicity, only individuals from China were included in our study. This is crucial as there have been reported variations in the risk of ischemic stroke and RAO among different ethnic groups. It is also important to acknowledge certain limitations in this study. First, longitudinal analysis of the association between changes in FGF21 levels and RAO was not possible due to the limited measurement of FGF21 levels at baseline. Second, the duration for detecting visual changes in patients with RAO ranged from 3 to 7 days. Considering the individual variability in recovery time which may lead to suboptimal vision at discharge, we conducted a one-month follow-up period for visual acuity correction. However, many patients were lost to follow-up, resulting in a lack of follow-up information for some patients. Moreover, the incidence of RAO is relatively low. Though we have made efforts to gather as many RAO patients from our hospital as possible, patients from other hospitals were not included, which may lead to bias in the research results or may not apply to other medical institutions.

In conclusion, our study emphasized that FGF21 could serve as a potential biomarker for the occurrence of RAO, but also highlighted the association between elevated FGF21 levels and worse visual outcomes and risk of concurrent ischemic stroke. Multi-center and international studies are needed to verify our findings and to investigate the molecular mechanism of FGF21 in RAO patients. Our study may provide a clinical basis for further application of FGF21 in the diagnosis and treatment of RAO patients.

### Supplementary Information

Supplementary Information.

## Data Availability

The data used and/or analyzed during the current study available from the corresponding author on reasonable request.
