# Supplementary material for: Circulating serum fibroblast growth factor 21 as risk and prognostic biomarker of retinal artery occlusion
Source: Sci Rep. 2024 May 24;14:11854. doi: 10.1038/s41598-024-62588-w (PMC11126651; doi:10.1038/s41598-024-62588-w)
Supplement: Supplementary file 1 — Supplementary Information. [file 41598_2024_62588_MOESM1_ESM.docx]

**Supplementary Table 1.** Correlates of FGF21 levels in multivariable linear regression analysis.

| Variables | Univariate analysis | | | | multivariate analysis | | | |
| --- | --- | --- | --- | --- | --- | --- | --- | --- |
|  | Unadjusted  coefficient | Standardized  coefficient | 95%CI | P | Unadjusted  coefficient | Standardized  coefficient | 95%CI | P |
| Male sex | 9.022 | 0.028 | -34.912 to 52.957 | 0.686 |  |  |  |  |
| Age | 0.639 | 0.038 | -1.653 to 2.931 | 0.583 |  |  |  |  |
| hypertension | 4.282 | 0.015 | -34.689 to 43.253 | 0.829 |  |  |  |  |
| Diabetes | -39.443 | -0.071 | -115.251 to 36.365 | 0.306 |  |  |  |  |
| WBC | 7.594 | 0.086 | -4.506 to 19.694 | 0.217 |  |  |  |  |
| Neu | 11.437 | 0.090 | -6.046 to 28.920 | 0.199 |  |  |  |  |
| Lym | 5.058 | 0.025 | -22.466 to 32.581 | 0.718 |  |  |  |  |
| NLR | 9.867 | 0.052 | -16.450 to 36.184 | 0.461 |  |  |  |  |
| Mono | 21.357 | 0.036 | -60.612 to 103.326 | 0.608 |  |  |  |  |
| ALT | 1.138 | 0.143 | 0.050 to 2.227 | 0.040 | 0.960 | 0.120 | -0.144 to 2.064 | 0.088 |
| AST | 1.056 | 0.125 | -0.098 to 2.210 | 0.073 |  |  |  |  |
| ALT/AST | 27.577 | 0.075 | -22.782 to 77.883 | 0.281 |  |  |  |  |
| TG | 57.998 | 0.290 | 31.635 to 84.362 | <0.001 | 43.937 | 0.220 | 15.331 to 72.544 | 0.003 |
| Tch | 12.967 | 0.072 | -11.919 to 37.853 | 0.305 |  |  |  |  |
| HDL-Ch | -142.570 | -0.273 | -211.688 to -73.452 | <0.001 | -78.935 | -0.151 | -153.005 to -4.864 | 0.037 |
| LDL-Ch | 25.739 | 0.128 | -1.646 to 53.123 | 0.065 |  |  |  |  |
| Urea | 0.988 | 0.010 | 12.286 to 14.261 | 0.883 |  |  |  |  |
| eGFR | -1.780 | -0.166 | -3.240 to -0.320 | 0.017 | -2.055 | -0.191 | -3.484 to -0.625 | 0.005 |
| Glu | 16,770 | 0.138 | 0.186 to 33.353 | 0.048 | 0.507 | 0.004 | -16.350 to 17.364 | 0.953 |

WBC: white blood cell; Neu: neutrophil; Lym: lymphocyte; NLR: neutrophil-to-lymphocyte ratio; Mono: monocyte; ALT: glutamic pyruvic transaminase; AST: glutamic oxaloacetic transaminase; ALT/AST: glutamic pyruvic transaminase-to -glutamic oxaloacetic transaminase ratio; TCh: total cholesterol; TG: triglycerides; HDL-Ch: high-density lipoprotein cholesterol; LDL-Ch: low-density lipoprotein cholesterol; eGFR: estimated glomerular filtration rate; Glu: glucose.

**Supplementary Table 2.** Logistic regression analysis of ln-transformed serum FGF21 level for RAO.

| FGF21  quartile | N | OR（95%CI） | | | |  |
| --- | --- | --- | --- | --- | --- | --- |
|  |  | Crude | Model 1 | | Model 2 | |
| All | 212 | 3.184(1.998, 5.071) | 3.202 (2.001, 5.125) | | 2.608 (1.367, 4.975) | |
| P for trend |  | <0.001 | <0.001 | 0.004 | |  |

Crude: no adjustment.

Model 1: Adjusted for sex, age, hypertension and diabetes.

Model 2: Adjusted for the same variables as Model 1 as well as Neu, Lym, NLR, Mono, AST, TG, HDL, eGFR and Glu.


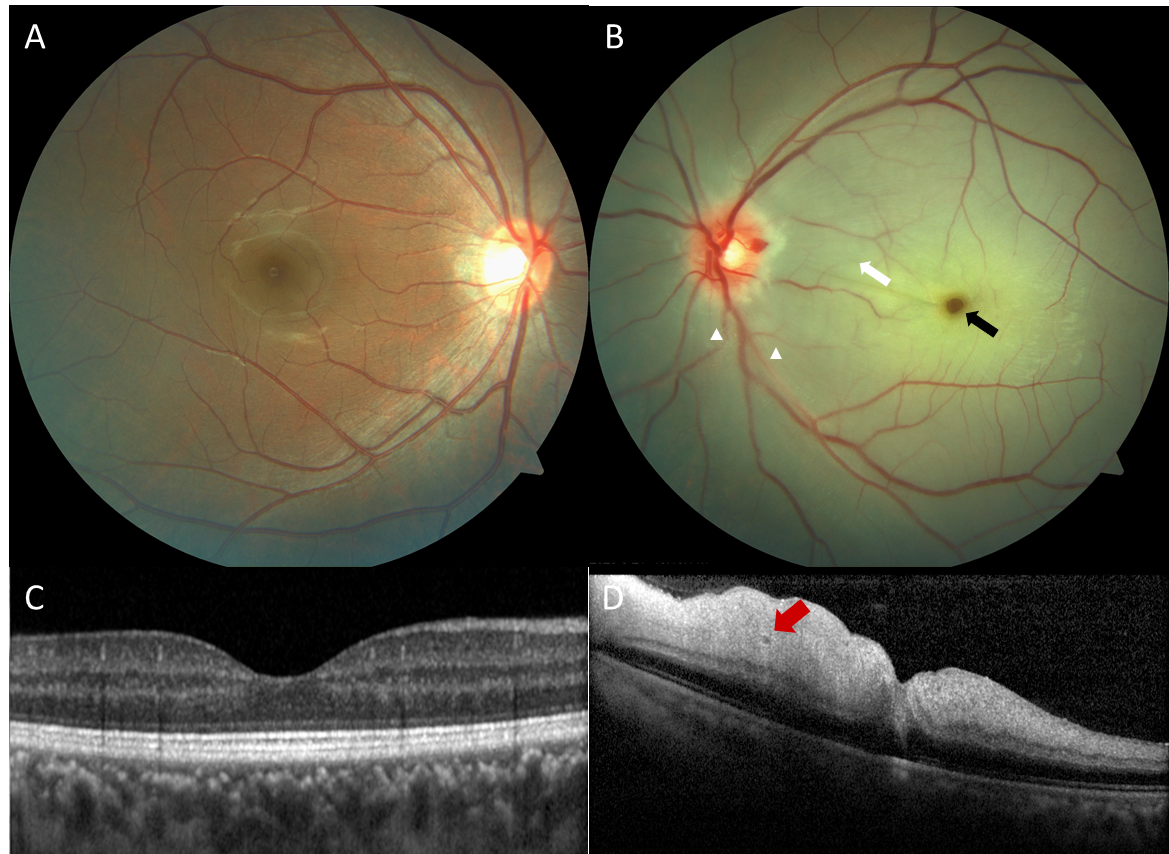


**Supplementary Figure 1.** Imaging **examination in acute central retinal artery occlusion (CRAO; left eye).** **A,** Fundus photograph of the normal right eye. **B,** Fundus photograph of the left CRAO showing diffuse retinal whitening (white arrow) with a cherry red spot (black arrow), attenuated retinal vessels (white triangle). **C,** Normal optical coherence tomography of the right eye. **D,** Optical coherence tomography of the left eye with acute CRAO demonstrates acute inner retinal edema (red arrow).

**
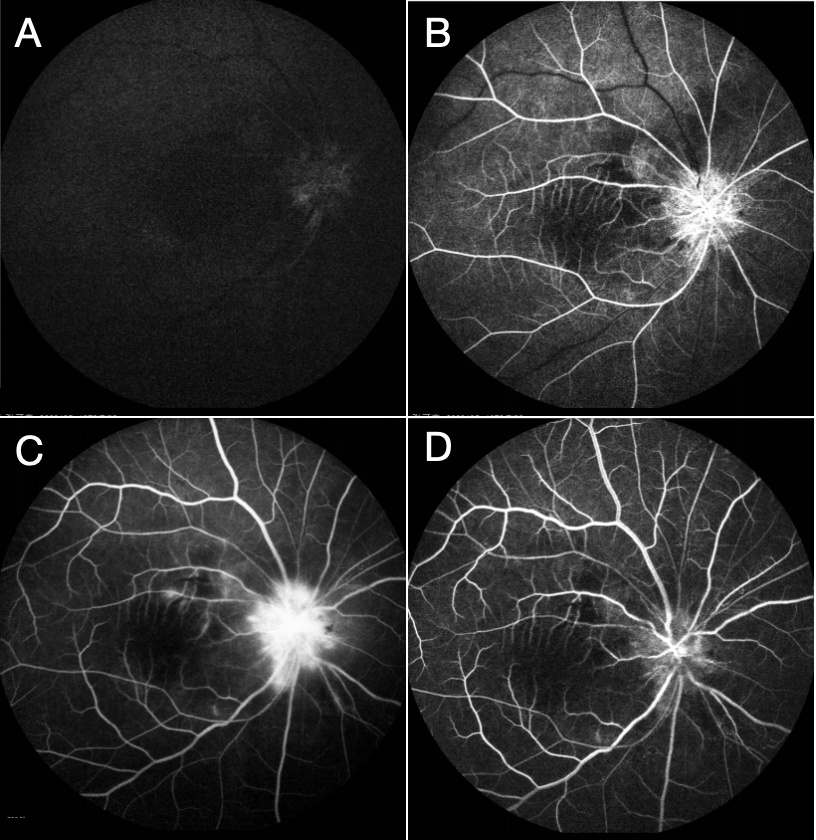
**

**Supplementary Figure 2.** Fluorescein funds angiography (FFA) **in acute central retinal artery occlusion (CRAO; right eye). A, Arterial phase. B, Arteriovenous phase. C, Venous phase. D, late phase.**


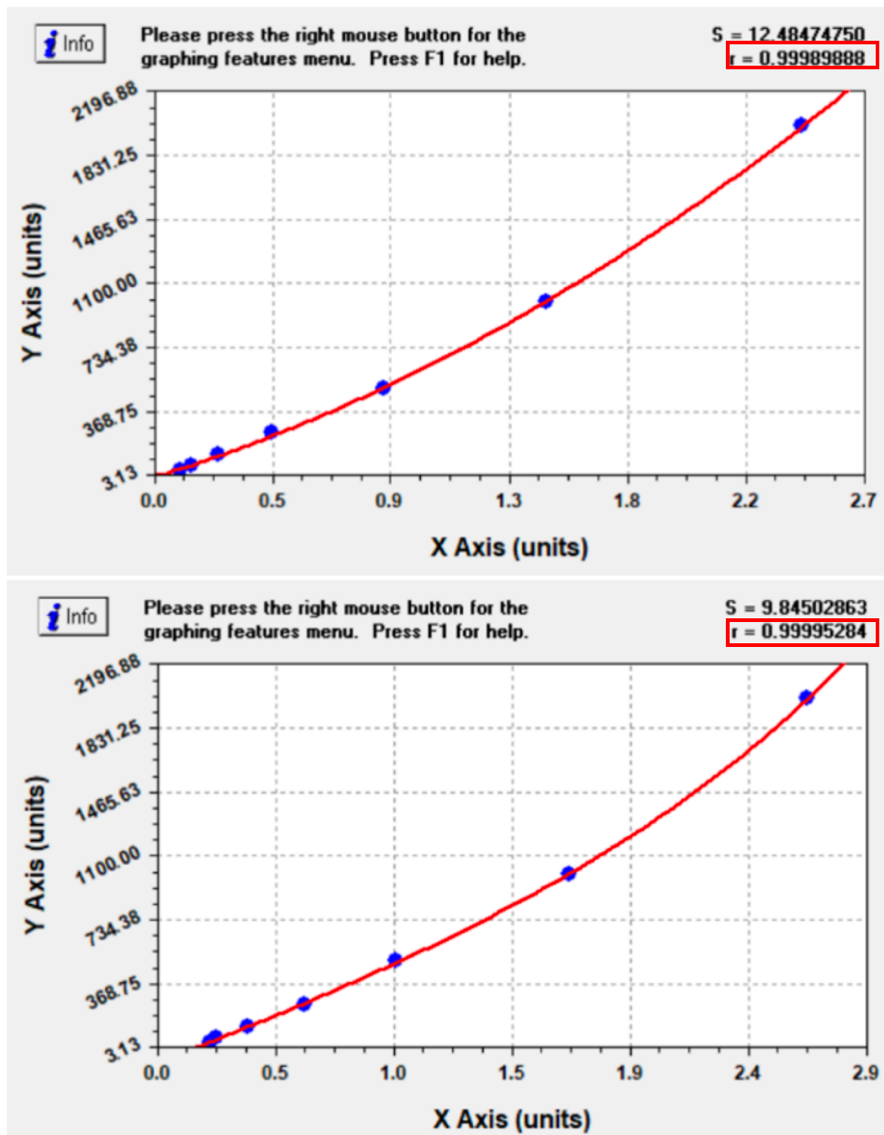


**Supplementary Figure 3.** Standard curve of different batches serum FGF21 assay plates. X Axis: absorbance; Y Axis: FGF21(pg/ml).
